# Application of Negative Pressure Wound Therapy with Instillation and Dwell Time of the Open Abdomen: Initial Experience

**DOI:** 10.7759/cureus.5667

**Published:** 2019-09-16

**Authors:** Luis G Fernandez, Pablo Sibaja Alvarez, Mark J Kaplan, Alfredo A Sanchez-Betancourt, Marc R Matthews, Alan Cook

**Affiliations:** 1 Surgery, Trauma Wound Care, University of Texas Health Science Center, Tyler, USA; 2 General Surgery, Universidad Federada San Judas Tadeo, San Jose, CRI; 3 Surgery, Einstein Medical Center, Philadelphia, USA; 4 Medical Administration, Hospital Mexico, San Jose, CRI; 5 Surgery, Arizona Burn Center, Phoenix, USA; 6 Epidemiology and Biostatistics, University of Texas Health Science Center, UT Health East Texas, Tyler, USA

**Keywords:** open abdomen, negative pressure wound therapy, wound healing, abdominal cavity, peritoneal lavage

## Abstract

Recent therapeutic advances in the management of severe abdominal sepsis (SAS) have improved patient mortality and morbidity. However, SAS and its impact on multiple organ failure remain a serious, life-threatening condition with a high mortality rate. The open abdomen (OA) technique has become an effective alternative to repeat laparotomy. The use of OA negative pressure wound therapy (OA NPWT) has been a significant advancement in the management of the open abdomen. Similarly, negative pressure wound therapy (NPWT) with instillation and dwell time (NPWT-i) has been used in patients with multiple comorbidities, with an American Society of Anesthesiology Classification ≥ 2, severe traumatic wounds, diabetic foot infections, and wounds complicated by invasive infection or extensive biofilm. Controlled instillation of saline during NPWT-i may further enhance healing by facilitating automatic and contained volumetric wound irrigation and cleansing and diluting local levels of inflammatory cytokines, improving the local as well as the systemic response to infection. Although the soft tissue and intra-abdominal compartments differ anatomically, they share very similar biologic responses to infections. Therefore, from a biologic and physiologic aspect, intraperitoneal instillation therapy may play a role as an adjunctive treatment of abdominal compartment inflammation from trauma or infection. The addition of saline solution instillation to OA NPWT (OAI) in a programmed, controlled manner may offer the clinician an effective adjunctive therapy for the treatment of the complex septic abdomen. The technical aspects of instillation into the OA and a pooled multicenter case study cohort utilizing OAI with saline solution, bacitracin, or hypochlorous acid in the management of the septic abdomen is presented.

## Introduction

The open abdomen (OA) technique using temporary abdominal closure (TAC) has been shown to be beneficial in the care of patients with complex abdominal pathology [1-3]. The TAC approach allows for a step-by-step approach to address the underlying pathologic process, offering patients the opportunity to recover from the initial shock state and achieve a level of physiologic homeostasis and allowing further surgical repair in a more controlled environment.

Early methods of TAC were passive and provided coverage for the abdominal contents; however, these techniques failed to adequately address intra-abdominal fluid/lymph and ongoing inflammation. The application of OA negative pressure wound therapy (OA NPWT) as a form of TAC (OA NPWT, Abthera™ Open Abdomen Negative Pressure Therapy, KCI, an Acelity Company, San Antonio, TX, US) has provided the surgeon with a more technically advanced device to assist in the care of patients with a complex abdominal pathology [4-5]. With the ability to remove intraperitoneal fluid while limiting lateral abdominal wall retraction (and in many cases, aiding in medial abdominal wall closure), these innovative forms of TAC represent dynamic systems that have improved overall patient outcomes and have become the preferred mode of TAC in patients [6].

Abdominal washouts (direct fluid irrigation into the peritoneal cavity) have been an integral part of the total management of the OA and has been used to help remove bacteria and other inflammatory mediators. However, washouts typically require a trip to the operating room (OR), which may not be feasible in some critically ill OA patients. Therefore, having a contained instillation system may provide a better option. Adding the instillation of a biocompatible solution to negative pressure wound therapy (NPWT-i), in a controlled manner, may offer the clinician an additional tool for the management of the complex septic abdomen.

We describe an easy-to-use method of intermittent fluid instillation within the open abdomen that effectively distributes fluid throughout the peritoneal cavity and the visceral contents. This technique incorporates standard negative pressure therapy and an additional set of tubing to allow for the flow of fluids. TAC with intra-abdominal fluid instillation may offer several advantages, over other modalities of TAC with repeated multiple laparotomies, in the management of the open abdomen.

## Materials and methods

Study design

This retrospective small, pooled case series, included seven patients with severe abdominal sepsis. OA instillation (OAI) was utilized in the treatment of all patients. The type of fluid instilled (saline solution, bacitracin, or hypochlorous acid) in each patient was selected by the treating physician.

During the intraoperative assessment, each abdomen was classified according to the amended OA classification system (Table 1) [7]. Duodenal and liver injury were graded according to the American Association of Surgery for Trauma (AAST) Organ Injury Scale (Table 2 and Table 3, respectively) [8]. The Hinchey scale for perforated diverticulitis was also used (Table 4) [9].

**Table 1:** Classification of the open abdomen Adapted from Björck et al. [7]

Grade	Description
1A	Clean open abdomen without adherence between the bowel and abdominal wall or fixity
1B	Contaminated open abdomen without adherence between the bowel and abdominal wall or fixity
1C	Enteric leak without fixation
2A	Clean open abdomen developing adherence between the bowel and fixity
2B	Contaminated open abdomen developing adherence between the bowel and fixity
2C	Enteric leak developing fixation
3A	Clean, frozen abdomen
3B	Contaminated, frozen abdomen
4	Established enteroatmospheric fistula, frozen abdomen

**Table 2:** Duodenum injury scale Adapted from Tinkoff et al. [8]

Grade	Damage	Injury Description
I	Hematoma	Single portion of the duodenum
	Laceration	Partial-thickness injury without perforation
II	Hematoma	More than one portion
	Laceration	Disruption <50% of circumference
III	Laceration	Disruption 50-75% of the circumference of the second portion
		Disruption of 50-100% of the circumference of the first, third, and fourth portion
IV	Laceration	Disruption >75% of the circumference of the second portion
		Involving ampulla or distal common bile duct
V	Laceration	Massive disruption of the duodenopancreatic complex
	Vascular	Devascularization of duodenum

**Table 3:** Liver injury scale Adapted from Tinkoff et al. [8]

Grade	Damage	Injury Description
I	Hematoma	Subcapsular, non-expansive, <10% of surface
	Laceration	Non-bleeding, <1 cm deep
II	Hematoma	Subcapsular, non-expansive, 10-50% of surface
	Laceration	1-3 cm deep, <10cm in size
III	Hematoma	Subcapsular, expansive >50% of surface or intraparenchymal >2 cm
	Laceration	>3 cm deep
IV	Hematoma	Bleeding intraparenchymal rupture
	Laceration	Involving 25%- 50% of lobe
V	Laceration	Parenchymal, involving more than 50% of lobe
	Vascular	Juxtahepatic vein, main hepatic veins, or retrohepatic cava
VI	Vascular	Hepatic avulsion

**Table 4:** Perforated diverticulitis scale Adapted from Hinchey et al. [9]

Grade	Description
I	Pericolic abscess
II	Pelvic, intraabdominal, or retroperitoneal abscess
III	Generalized purulent peritonitis
IV	Generalized fecal peritonitis

Damage control surgery

Damage control surgery was performed on all patients following principles, as originally described by Rotondo et al., for the resuscitation of trauma patients [10]. The component steps of DCS are as follows:

• Part zero (DC 0): Early/rapid, severe injury pattern recognition for potential damage control candidates.

• Part one (DC I) Immediate exploratory celiotomy with rapid control of bleeding, contamination, abdominal packing, and temporary wound closure in the operating room.

• Part two (DC II): Controlled resuscitative phase for physiological and biochemical stabilization) and a thorough tertiary examination to identify all injuries in the intensive care unit (ICU).

• Part three (DC III): Re-exploration to perform definitive repair of all injuries, occurring once physiology has normalized.

Open abdomen instillation

**Figure 1:**
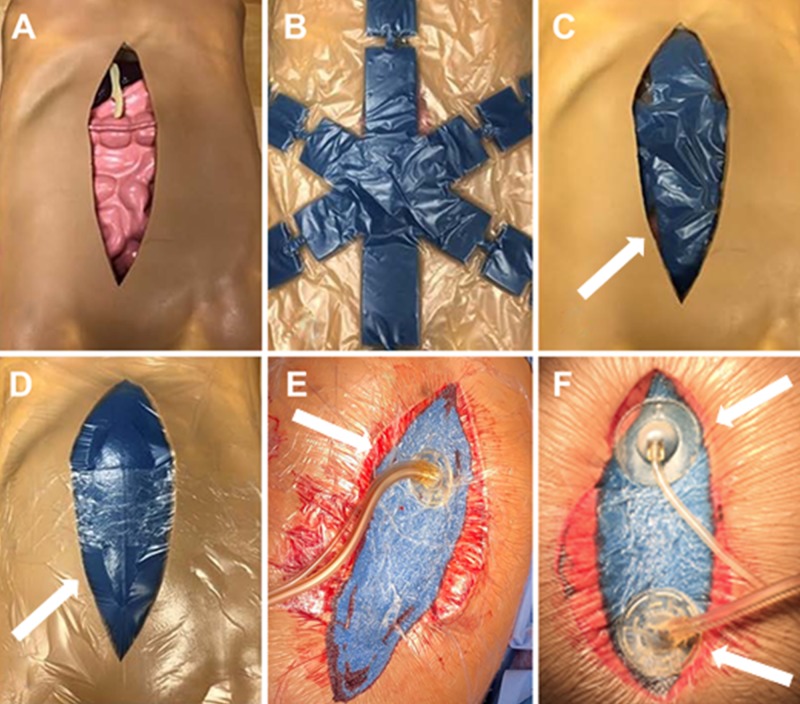
Application of open abdomen instillation A. Open abdomen; B. Visceral protective layer; C. Visceral protective layer placed over the abdominal contents in the abdominal cavity; D. Intraabdominal placement of perforated foam dressing; E. Placement of dual lumen port tube set; F. Placement of the double port tube set. Adapted from Matthews et al. [11]

OAI is initiated in the operating room (or, if needed, at the patient’s bedside, in the surgical intensive care unit (ICU)). Application of OAI has been previously described [11]. Briefly, following abdominal washout, the OA fenestrated visceral protective layer (Abthera™ Fenestrated Visceral Protective Layer, KCI, an Acelity Company) is cut to size (as needed), placed over the abdominal contents, and the edges tucked into the paracolic gutters (Figures 1A-1C). The perforated foam dressing (Abthera™) is then placed directly on top of the visceral protective layer followed by the application of drape (Abthera™) ensuring an overlap of at least 8 cm over the periwound skin (Figure 1D). A dual lumen port (V.A.C. VeraT.R.A.C.™ Pad, KCI) or double port tubing set (V.A.C. VeraT.R.A.C. DUO™) is then placed over the occlusive foam dressing for the delivery of negative pressure and the instillation of a biocompatible solution (Figures 1E-1F). The double port tubing set contains two pads for larger or vertical wounds that provide for the individualized delivery of negative pressure and the instillation solution. This allows for customized pad placement to enhance fluid circulation and removal. The tubing set is connected to the fully automated NPWT-i unit (V.A.C. Ulta™ Therapy unit, KCI) to provide both controlled instillation and aspiration of the OA instillation solution.

An additional fluid container of one liter of the instillation solution should be connected and replaced, as needed, depending on the treatment parameters. The holding canisters should also be replaced as often as required to guarantee that the OA instillation can be properly delivered. Suggested treatment parameters are described in Table 5.

**Table 5:** Suggested treatment parameters

Category	Suggested Parameter
Pressure setting	100 - 125 mmHg
Instillation volume	200 - 300 mL (per cycle)
Dwell time	20 - 30 minutes
Negative pressure cycle time	120 minutes
Total treatment cycle time	140 - 160 minutes
Total cycles per day	10

## Results

Patient 1: irrigation of a grade 3B open abdomen

**Figure 2:**
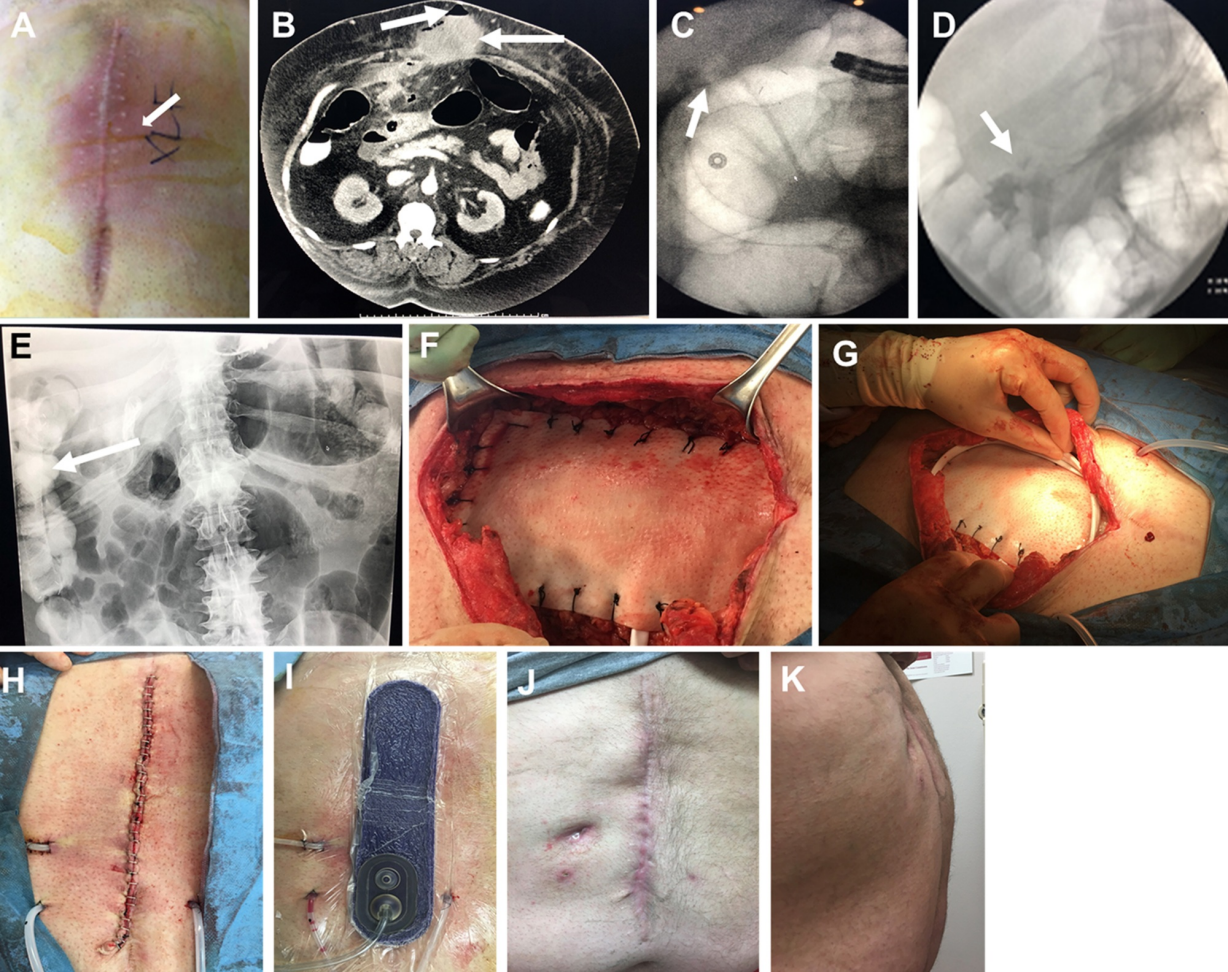
Open abdomen repair and instillation of a contaminated, frozen abdomen A. Midline erythema; B. Abdominal CT showing potential bowel perforation (left arrow) and abdominal abscess (right arrow); C. Endoscopic duodenal stent placement; D. Elimination of duodenal leak achieved (arrow); E. Duodenal stent in right colon (arrow); F. Application of reconstruction tissue matrix; G. Drain application; H. Closed abdominal incision, I. Application of closed incision negative pressure therapy; J. Anterior view of fully healed abdominal incision; K. Lateral view of fully healed abdominal incision

A 57-year-old, morbidly obese male presented to the emergency department for the evaluation of acute abdominal pain, with a 10-cm area of induration, erythema, tenderness, and swelling of the midline of the abdomen (Figure 2A). The patient had a history of exploratory laparotomy for a perforated duodenal ulcer within the last 10 months with a Graham Patch repair. Laboratory blood tests revealed elevated white blood cell (WBC) counts. An abdominal computed tomography (CT) scan with intravenous (IV) contrast was performed and indicated a possible bowel perforation with anterior abdominal wall abscess (Figure 2B). The patient was taken to surgery. Operative findings included a mesogastric incisional hernia containing a portion of the transverse colon, a deep right upper quadrant abscess tract, a small amount of enteric fluid, and frank purulent discharge. A limited exploratory celiotomy and debridement of the ventral hernia abscess were performed. A 7-mm closed suction drain was placed in the right upper quadrant directed towards the suspected duodenal fistula. Open abdominal irrigation was initiated using 1 L of hypochlorous acid wound cleansing solution (Vashe®, SteadMed®, Fort Worth, TX, US; off-label usage) with 10-minute dwell time. After the initial irrigation, a non-adhering silicone dressing (Adaptic Touch™ Non-Adhering Silicone Dressing, Systagenix, an Acelity Company, Gargrave, UK) was applied, and OAI initiated (instilling 200 cc normal saline with 20-minute dwell time, followed by two hours of negative pressure at -125 mmHg). The patient was admitted to the surgical/trauma unit and underwent intensive resuscitation. Upper gastrointestinal endoscopy revealed a perforated duodenal ulcer, and a covered stent was placed by the gastroenterologist (Figure 2C). Four days later, a follow-up CT scan revealed a small amount of residual air in the area of duodenal perforation; no abscess or bowel obstruction was noted. Prior to definitive closure, the patient underwent upper gastrointestinal fluoroscopic assessment, which showed no extravasation of contrast. The stent, which had been placed four days prior, had passed into the right colon (Figures 2D-2E). The patient was taken for definitive abdominal wall closure on hospital day (HD) six. Re-exploration of the abdominal cavity was performed with a 360-degree myocutaneous flap advancement. The abdomen underwent reconstruction using a 10 x 20 cm reconstructive tissue matrix (Strattice™ Reconstructive Tissue Matrix, LifeCell, an Allergan affiliate, Branchburg, NJ, US), utilizing an overlay technique followed by direct, manual irrigation of the incision using a hypochlorous acid wound cleansing solution (Figures 2F-2H). Bilateral subcutaneous drains were placed followed by skin closure using staples and the application of closed incision negative pressure therapy (ciNPT, Prevena™ Incision Management System, KCI, an Acelity Company, San Antonio, TX, US) (Figure 2I). The patient progressively improved and was discharged home on HD 12. At the postoperative follow-up (76 days post-discharge), the patient was asymptomatic and living a normal life (Figures 2J-2K).

Patient 2: perforated appendicitis with delayed presentation and septic abdomen, grade 2B open abdomen

**Figure 3:**
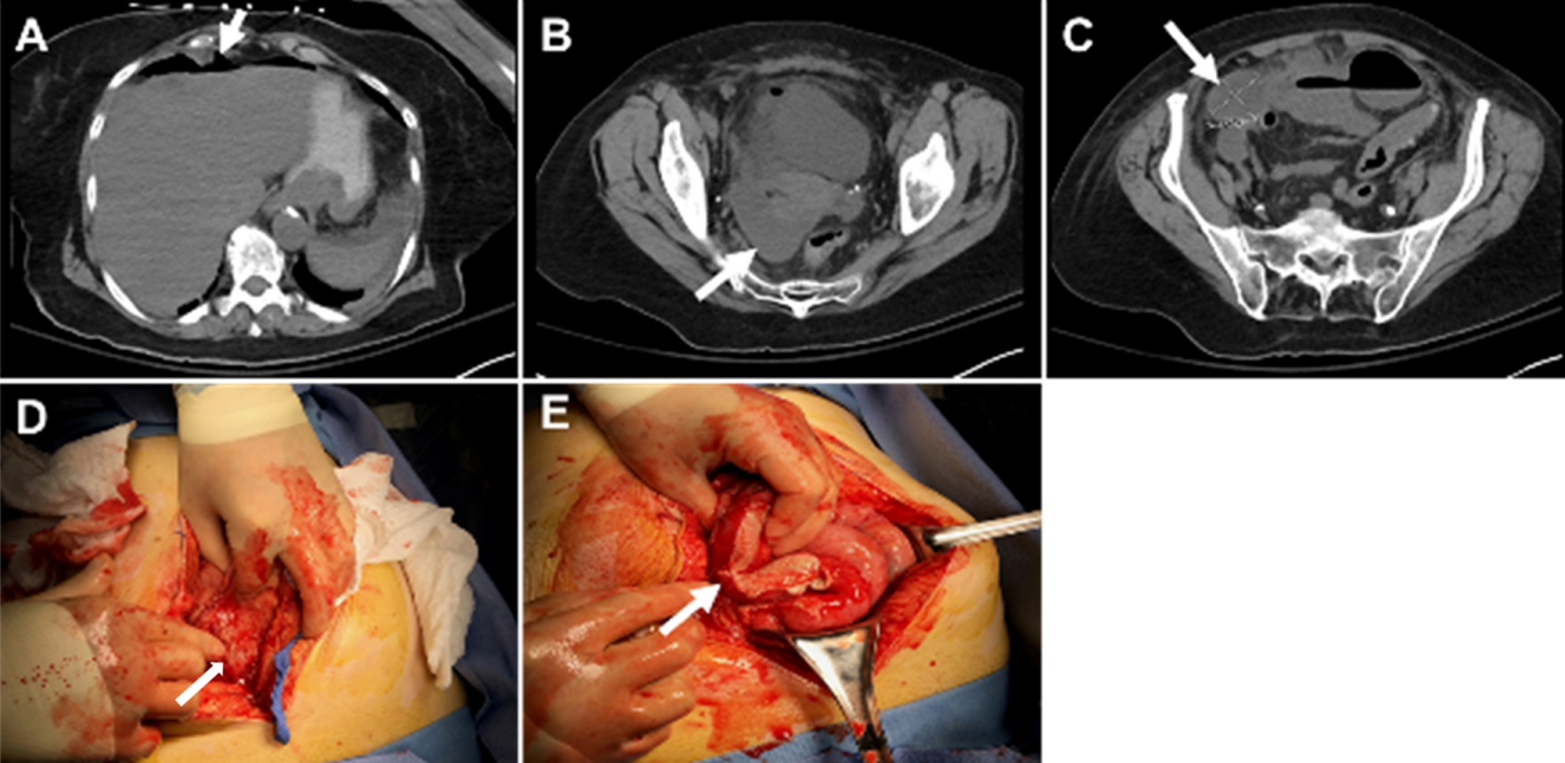
Perforated appendicitis with delayed presentation and septic abdomen, grade 2B open abdomen A. Pneumoperitoneum identified in the patient’s abdomen (arrow); B. Diffuse peritoneal fluid identified in abdominal CT scan (arrow); C. Diffuse peritoneal fluid identified in abdominal CT scan (arrow); D. Diffuse inflammation of bowel (arrow); E. Fibrin deposition involving the large and small bowel (arrow)

A 76-year-old female with dementia and acute encephalopathy was admitted to the hospital. On physical examination, the patient displayed diffuse abdominal pain and diffuse peritonitis. A CT scan of the abdomen with oral contrast revealed a large pneumoperitoneum and diffuse fluid within the abdominal cavity, consistent with a perforated viscus (Figures 3A-3C). The patient underwent exploratory laparotomy, adhesiolysis, aerobic/anaerobic cultures, drainage or debridement of a pelvic/inter-loop abscess, and abdominal irrigation with 2 L of hypochlorous acid wound solution (off-label usage) with 10-minute dwell time. A fibrin sealant (Tisseel, Baxter International Inc., Deerfield, IL, US) in the right upper and lower quadrants was added, followed by the placement of a mechanical bioresorbable adhesion barrier (Seprafilm, Sanofi-Aventis, Bridgewater, NJ, US) and initiation of NPWT-i (instillation of 200 cc normal saline with 20-minutes dwell time, followed by negative pressure therapy (-125 mmHg) for two hours). Intraoperative findings were consistent with perforated appendicitis with delayed presentation and a septic abdomen, Grade 2B (Figures 3D-3E). The patient was admitted to the intensive care unit for continued aggressive resuscitation. She was taken for abdominal washout and closure 48 hours after the index operation. The operating surgeon described no purulent fluid or fibrin deposition and minimal adhesions. There was evidence of an inflammatory reaction in the right lower quadrant, with normal-appearing large and small bowel. The patient underwent primary closure and continued to improve. The patient was discharged to a skilled nursing facility.

Patient 3: blunt traumatic grade III injury to the second portion of the duodenum and grade III blunt liver laceration septic abdomen, grade 2B open abdomen

**Figure 4:**
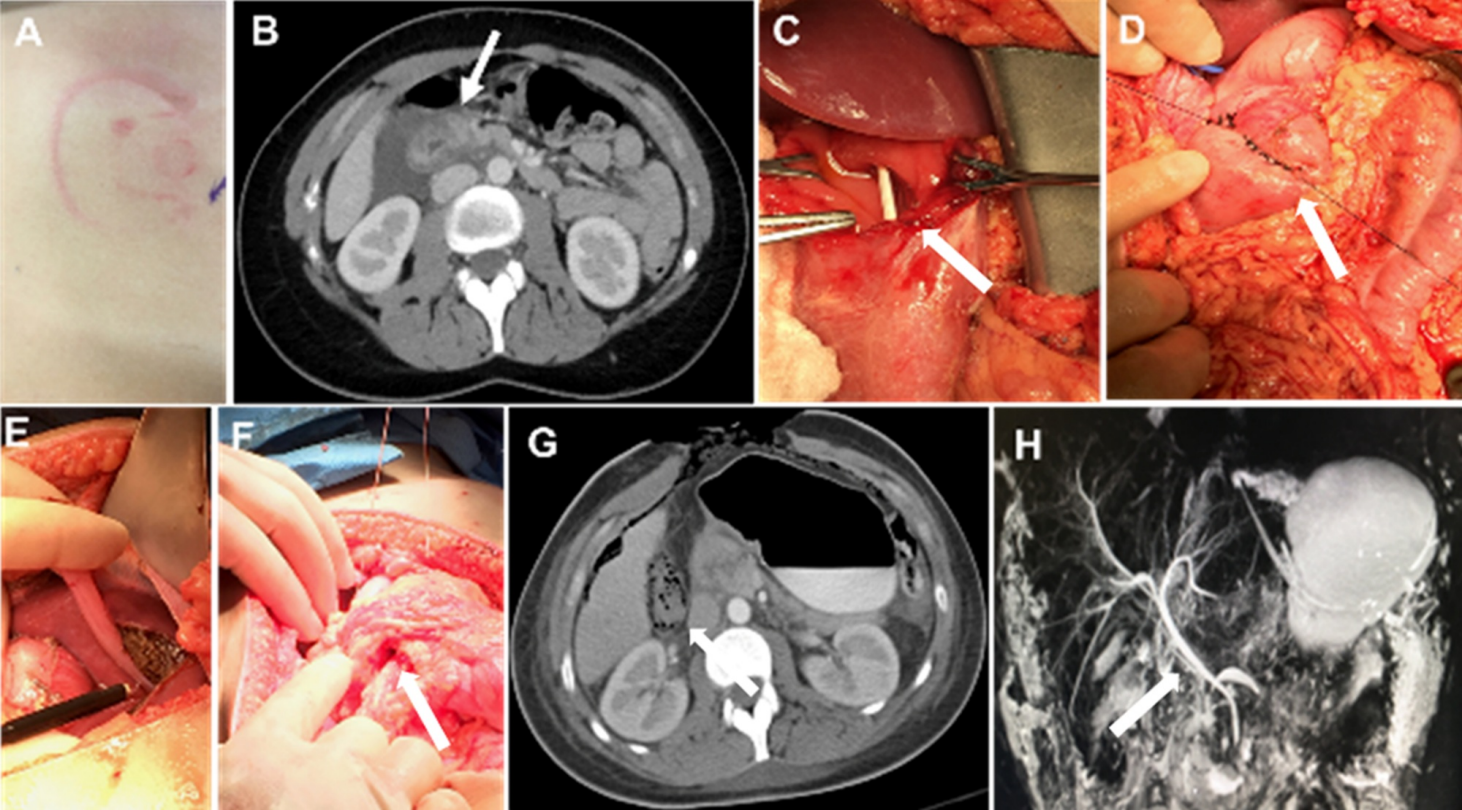
Blunt traumatic grade III injury to the second portion of the duodenum and grade III blunt liver laceration septic abdomen, grade 2B open abdomen A. Saddle pommel imprint; B. Edematous second portion of the duodenum with peri-duodenal fluid; C. Stenting of the duodenal defect (arrow); D. Two-layer primary duodenal repair (arrow); E. Repair of the liver laceration (arrow); F. Primary duodenal repair with Graham Patch (arrow); G. No duodenal leaks following repair (arrow); H. Intact hepatobiliary system following repair (arrow)

A 20-year-old female presented to the emergency department (ED) with back pain that began earlier when the patient was thrown off a horse and the horse landed on her. The pain was located primarily in the cervical and thoracic sections of her back. Associated symptoms include right-sided pelvic pain, abdominal pain, and rib pain. The abdominal pain was severe and was located in the upper and lower sections of her abdomen. Figure 4A illustrates the saddle pommel imprint on the patient’s right upper quadrant. A CT of the abdomen and pelvis with IV contrast was obtained and showed fluid around the duodenum, suggesting a possible duodenal rupture (Figure 4B). The patient was taken emergently for exploratory laparotomy. The patient underwent surgical exploration via a midline incision, a right Cattel -Brasch medial rotation with a Kocker maneuver, exploration of the lesser sac, debridement, and two-layer repair of the second portion of duodenum with Graham Patch placement, repair of the Grade III left lobe of liver laceration with the argon beam coagulator and round/falciform ligament patch, abdominal washout, placement of a fibrin sealant on the duodenal/liver repair (Figures 4C-4F), and placement of OA instillation and support lines. Instillation was initiated once the patient was in the surgical intensive care unit. Therapy parameters include the instillation of 200 cc normal saline with a dwell time of 20 minutes followed by negative pressure (-125 mmHg) for two hours. Before definitive closure, a CT of the abdomen and pelvis, as well as a magnetic resonance imaging (MRI) scan of the biliary system, was performed. No surgical lesions were identified (Figures 4G-4H).

On postoperative day three, the patient underwent exploratory laparotomy, abdominal washout, placement of a distal feeding jejunostomy tube, closed suction drain placement along the right upper quadrant, and primary closure of the abdominal wall. No evidence of a duodenal leak or significant bowel edema/inflammation was noted intraoperatively. Closed incision negative pressure therapy was applied over the closed incision and left in place for six days. Prior to the closure of the incision, bupivacaine was injected into the fascia and subcutaneous tissue to help manage postoperative pain. A fluoroscopic upper gastrointestinal contrast study was obtained on postoperative day six. No evidence of a leak from the duodenal repair was observed. The patient progressively improved, tolerating a general diet and ambulation, and was discharged home on HD 13.

Patient 4: gunshot wound to the abdomen (grade II injury)

A 26-year-old male presented with a gunshot wound to the abdomen. AAST Grade 2 sigmoid and colon injuries were observed during the abdominal assessment. The sigmoid and colon injuries were repaired, and the abdomen left open for 24 hours, utilizing the OA NPWT System as a TAC, followed by primary closure.

**Figure 5:**
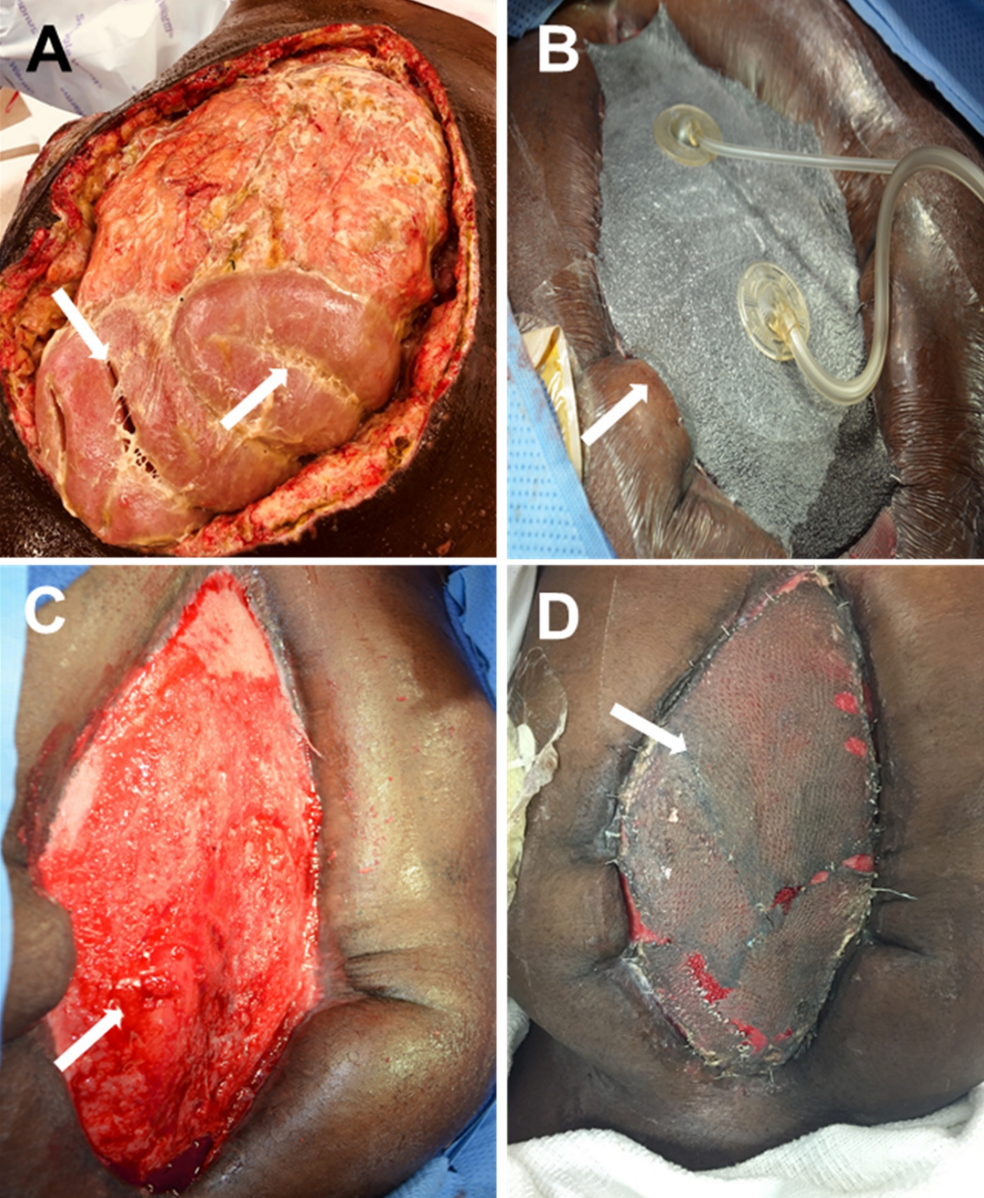
Gunshot wound to abdomen with Grade 2 injury to sigmoid and colon A. Intraoperative exudate and pockets of gross purulence; B. Application of open abdomen instillation; C. Granulation tissue developing over the dermal regeneration template; D. Abdomen five days after NPWT use over split-thickness skin graft

Ten days after surgery, the patient developed intra-abdominal sepsis secondary to an anastomotic breakdown with antibiotic-resistant Escherichia coli. Systemic antibiotics were initiated. The patient’s abdomen could not be closed secondary to bowel edema and infection (Figure 5A). Re-exploration surgery was performed, and OAI initiated. Normal saline (500 cc) and bacitracin (1 g) were instilled into the OA, followed by a 15-minute dwell time and four hours of continuous negative pressure (-125 mmHg) (Figure 5B). The patient underwent re-exploration surgery every 48 hours to drain the loculated pus and undergo dressing changes. After 10 days, the infection had resolved; however, the abdomen was unable to be closed, and a dermal regeneration template (Integra® Dermal Regeneration Template, Integra LifeSciences, Plainsboro, NJ, US) was placed over the peritoneal contents to stimulate the development of granulation tissue (off-label usage) (Figure 5C). After healthy granulation tissue covered the wound bed, the patient underwent a split-thickness skin graft to the abdominal wall. Traditional NPWT (V.A.C.® Therapy, KCI) was used over the graft and left in place for five days (Figure 5D). The patient was discharged from the hospital and is awaiting definitive abdominal wall closure.

Patient 5: grade III perforated colon and intra-abdominal sepsis

**Figure 6:**
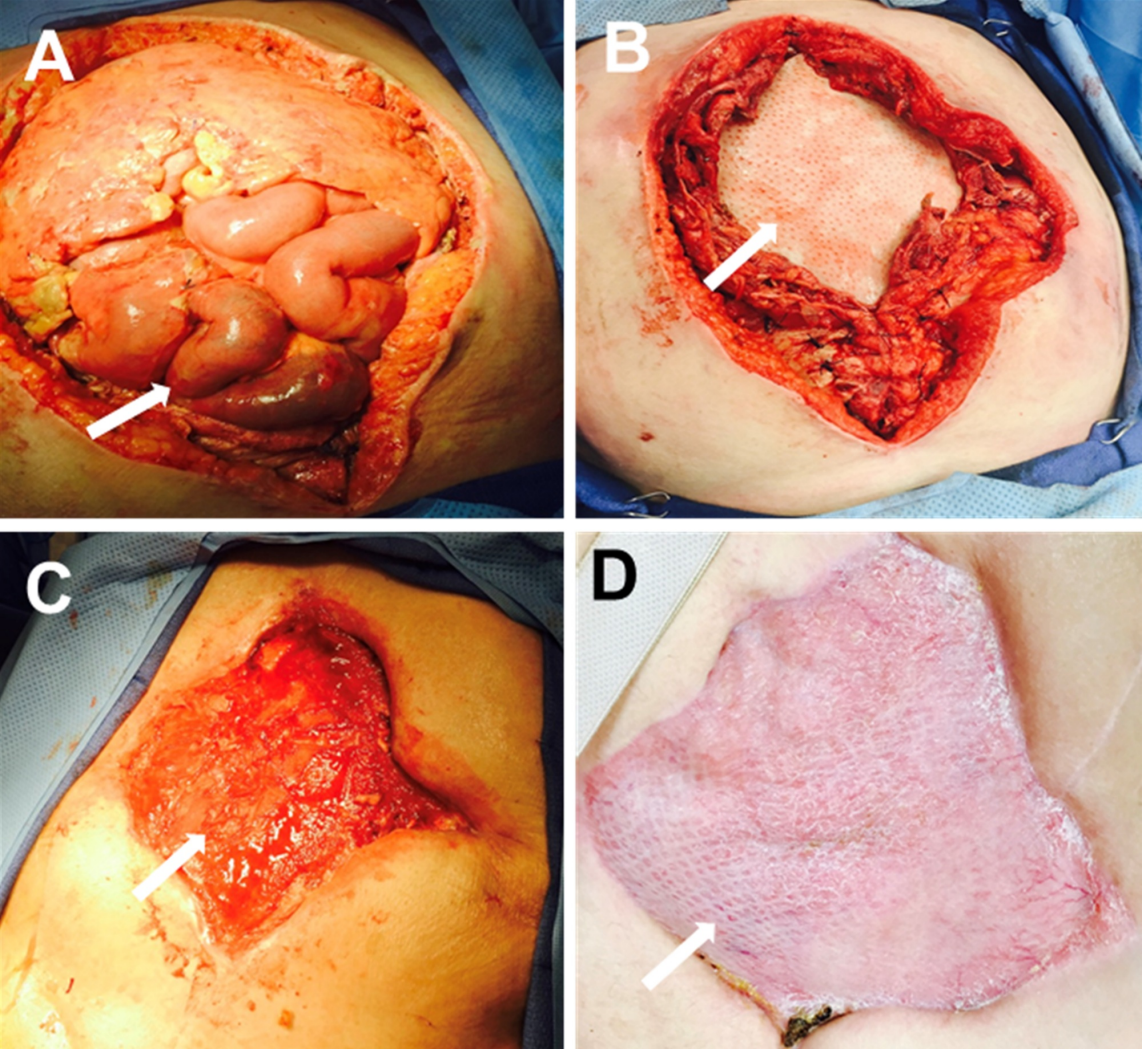
Perforated colon and intra-abdominal sepsis A. Open abdomen after five days of instillation therapy; B. Reconstructive tissue matrix placement; C. Granulation tissue development after two weeks of instillation; D. Application of split-thickness skin graft

A 65-year-old female presented with a perforated colon and intra-abdominal sepsis (Hinchey Grade III). Systemic antibiotics were given at presentation. Damage control surgery was performed with resection of the sigmoid colon. OAI was initiated at the time of the index surgery, with 500 cc normal saline and 1 g of bacitracin with a dwell time of 15 minutes, followed by four hours of continuous negative pressure (-125 mmHg). After five days of OAI, sepsis was controlled (Figure 6A). Due to massive bowel edema, the abdomen could not be closed, and a reconstructive tissue matrix was applied (Figure 6B). Instillation using NPWT-i on the abdominal wall wound was initiated (using 500 cc normal saline, 15-minute dwell time, and four hours of continuous negative pressure) to prevent desiccation and promote the formation of granulation tissue. Granulation tissue development was observed after 14 days of NPWT-i therapy (Figure 6C). A split-thickness skin graft was performed once the wound showed 100% coverage of granulation tissue (Figure 6D). Traditional NPWT was placed on the graft as a bolster for five days.

Patient 6: grade III perforated diverticulitis and diffuse peritonitis

**Figure 7:**
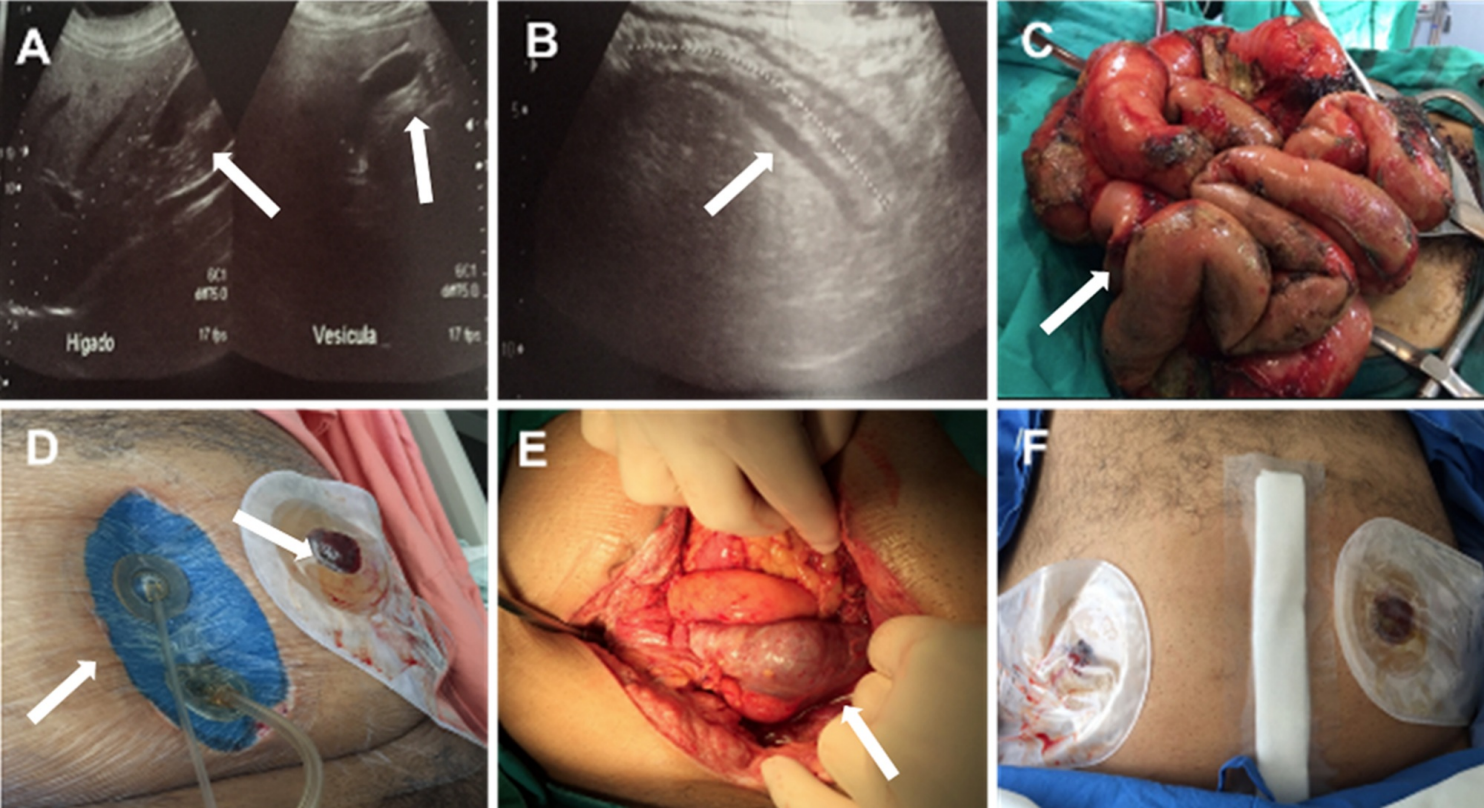
Perforated diverticulitis and diffuse peritonitis A. Abdominal ultrasound showing cholecystolithiasis (arrows); B. Abdominal ultrasound showing acute appendicitis (arrow); C. Active diffuse suppurative peritonitis; D. Abdominal installation and temporary colostomy; E. Open abdomen 72 hours after abdominal instillation (arrow); F. Closed abdomen

A 32-year-old morbidly obese male presented to the ED for evaluation due to a five-day lower abdominal pain, distension, and fever. Patient assessment showed signs of peritoneal irritation with a positive McBurney sign and tenderness of the pelvic region. Abdominal ultrasound reported cholecystolithiasis with a non-inflamed gall bladder and findings compatible with acute appendicitis without specific fluid collection (Figures 7A-7B). A Mannheim Peritonitis Index Score of 16 was calculated [12]. The patient underwent exploratory surgery. Active diffuse suppurative peritonitis with adhesions and multiple contaminated peritoneal fluid collections (OA Grade 2B) were observed (Figure 7C). Fluid samples were obtained for cultures. Extensive saline lavage and debridement were performed. A perforated sigmoid diverticulitis and inflamed sigmoid colon (Hinchey Grade III) was identified. A resection of the sigmoid and a temporary colostomy (Hartman’s’ Procedure) were performed [13].

Due to visceral edema, extensive contamination, and active peritoneal inflammation, a staged management treatment plan was initiated. OAI was initiated with 200 cc saline instillation followed by a 20-minute dwell time and two hours of continuous negative pressure at -125 mmHg (Figure 7D). Intravenous antibiotics, prophylactic anticoagulant medication, and analgesics were also given. At postoperative follow-up assessments, the patient showed reduced pain, increased appetite, decreased abdominal distention, and normalized vital signs. After 72 hours, the patient returned to the OR for abdominal exploration and surgical washout. The abdominal cavity was clean with reduced edema (Figure 7E), allowing for primary fascial closure in a non-tension environment with Penrose drain placement on the opposite pelvic side (Figure 7F). The abdominal closure procedure was performed without complications. Seven days after closure, the patient was discharged with no signs of active infection and oral tolerance to fluids and soft food.

Patient 7: perforated appendicitis with delayed presentation and septic abdomen (grade 2B)

**Figure 8:**
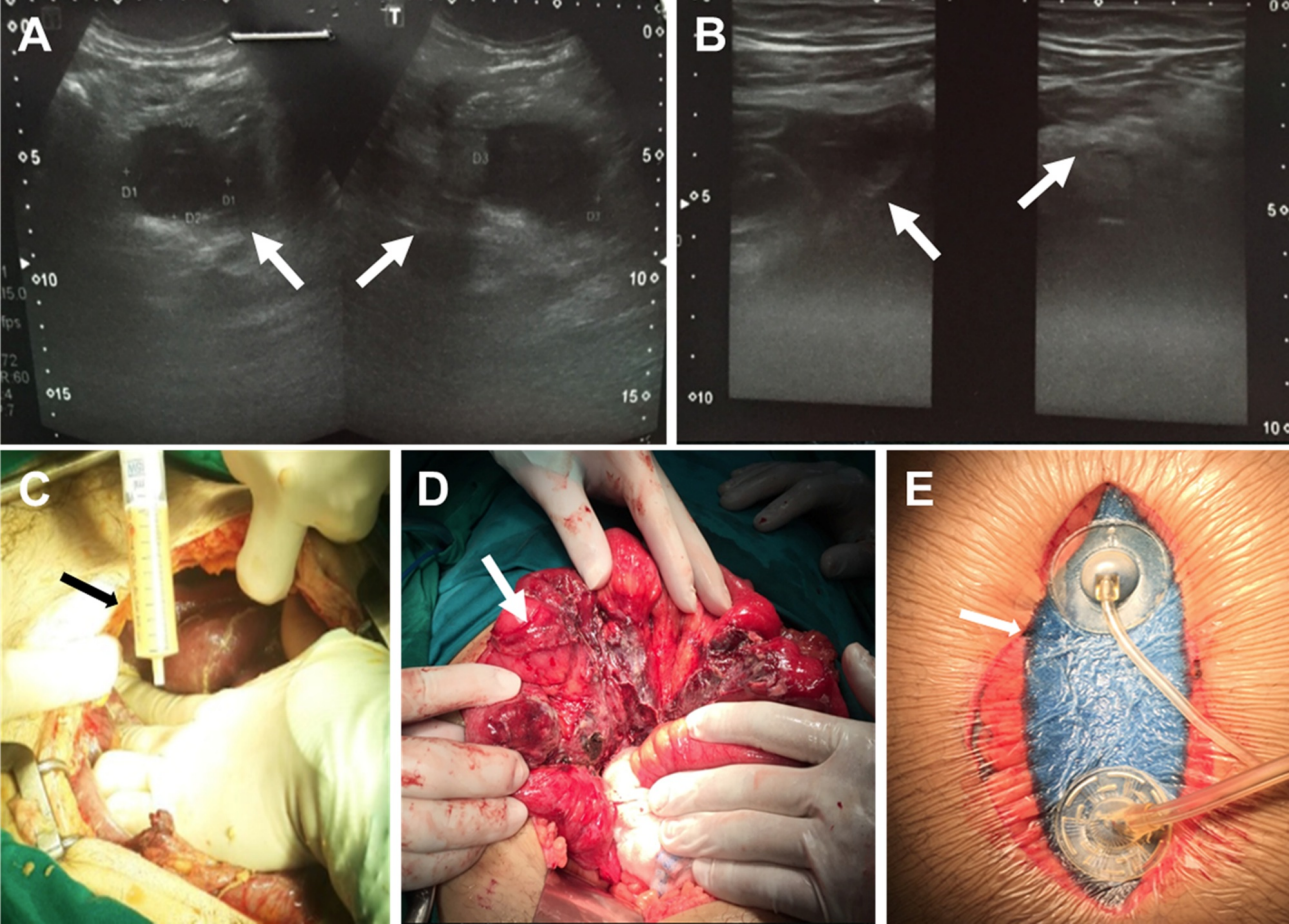
Perforated appendicitis with delayed presentation and septic abdomen (Grade 2B) A. Abdominal ultrasound showing a perforated appendix (view one, arrows); B. Abdominal ultrasound showing a perforated appendix (view two, arrows); C. Abdominal lavage; D. Placement of the ileum (arrow); E. Application of abdominal instillation

A 32-year-old male with no history of chronic disease presented to the ED for the second time in a five-day period, complaining of abdominal pain present for 10 days, oral intolerance, vomiting, and multiple foul-smelling liquid stools. Patient evaluation showed signs of dehydration, tachycardia, fever, and abdominal distension with abdominal rebound tenderness and induration of the right lower quadrant. An abdominal ultrasound showed concentrated non-mobile edematous small bowel and cecum aggregation in the right lower quadrant wrapping around a fluid collection with a calculated volume of 56 cc consistent with a retrocecal pelvic abscess displacing the bladder and rectum (Figures 8A-8B). A portion of a tubular thickened structured, possibly a portion of the bowel, adjacent to the fluid collection was described. The patient was diagnosed with a perforated retrocecal appendicitis with an interloop pelvic abscess and potential mechanical intestinal obstruction. The patient had a Mannheim Peritonitis Index Score of 21.

The patient underwent exploratory infra-umbilical midline laparotomy, adhesiolysis, aerobic/anaerobic cultures, drainage or debridement of the pelvic/inter-loop abscess, appendectomy, and extensive saline lavage. Intraoperative findings were consistent with perforated appendicitis with retrocecal interloop abscess and mechanical intestinal obstruction and a septic abdomen (OA Grade 2B) (Figures 8C-8D). OAI was initiated using 200 cc saline with a 20-minute dwell time, followed by two hours of continuous negative pressure at -125 mmHg. Intravenous antibiotics were given. After three days, the patient was taken to the OR for abdominal washout. During the second operation, no residual purulent fluid or fibrin deposition was observed and minimal adhesions required some visceral displacement. The persistence of some fluid warranted a prolonged OAI approach for a second 72-hour term (Figure 8E). Three days after the re-exploration surgery, the patient returned to the OR for surgical abdominal washout, definitive closure, and drain placement. Seven days after primary closure, the patient was discharged from the hospital in good condition.

## Discussion

Historically, surgeons were trained to perform a definitive operation for a patient with severe abdominal trauma, sepsis, hemorrhage, or other conditions, as an open abdomen following laparotomy was considered sub-optimal treatment. However, the closure of the abdomen sometimes occurred too quickly, which often led to complications and even death [3,14]. This initial multicenter, pooled case series serves as a first look into the applicability of combining the TAC technique using NPWT with OA instillation performed by different surgeons in a variety of clinical settings.

The addition of fluid instillation to traditional OA NPWT has been previously reported as a valid method of managing abdominal sepsis in patients requiring an OA approach [11,15-18]. D’Hondt et al. reported on the instillation of an antibiotic solution following pancreatic surgery using OAI, concluding that OAI was a promising adjunctive treatment when traditional therapy fails to manage the infection [15]. A 12-patient pilot study by Jimenez-Fuerteset al. and a successful case by Nisi et al. showed similar results in patients with abdominal sepsis or secondary septic peritonitis with OAI using saline [16-17]. In the largest case series to date, Sibaja et al. reported higher fascial closure rates, displayed lower mortality rates, and reduced hospital and ICU length of stay without complications resulting from OA instillation following the use of OAI in 48 patients with severe abdominal sepsis [19]. In our study, the use of OAI with normal saline, bacitracin, or hypochlorous acid solutions, in addition to damage control surgery, allowed for abdominal closure without complications.

One potential clinical concern for OAI is the possibility of hypothermia. A recent Cochrane meta-analysis review assessed inadvertent perioperative hypothermia (a drop in core temperature to below 36°C) [20]. According to this review, there appears to be a link between the instillation of fluid into the abdominal cavity and hypothermia. The investigators reported no statistically significant differences in core body temperature or shivering between individuals given warmed and room temperature irrigation fluids [20]. In our study, no evidence of hypothermia as a meaningful clinical complication was found with a low (5 L) or high volume (25 L) of direct abdominal irrigation. However, it is important that clinicians closely observe their patients during fluid instillation to ascertain the presence of reduced body temperature as a potential complication of the OA and intraabdominal fluid instillation.

Many types of instillation fluids have been utilized in the abdominal cavity with different results. Antibiotics have been regularly used as part of the instillation of the abdomen [21]. While many studies have been conducted with regards to their use in this context, the benefits of adding antibiotic solutions to the septic abdominal cavity remain unclear, and its pharmacological-clinical effectiveness remains unknown [22]. Other types of solutions such as anesthetics, more commonly, bupivacaine, have been utilized as post-surgical analgesia with varying levels of success [23-24]. Hypochlorous acid is also utilized as an instillation fluid to reduce abdominal fluid viscosity, thus increasing the removal of inflammatory ascites and septic material in the abdominal cavity [25]. There is little evidence of which instillation fluid is superior. While there are studies in which the use of antibiotics in the instilled solutions appears to yield better results, other studies fail to show the added benefit. The same case can be made for the use of anesthetics, which also fails to provide consistently superior pain management.

## Conclusions

Recent therapeutic advances in the management of OA have improved patient mortality and morbidity. This case series suggests that the OA NPWT technique is an effective alternative to repeated multiple laparotomies in patients with diffuse abdominal sepsis. The addition of saline solution or hypochlorous acid solutions to OA NPWT in a programmed, controlled manner may offer the clinician an effective adjunctive therapy for the treatment of the complex septic abdomen. The use of OAI in these seven patients resulted in abdominal closure while minimizing septic complication in all patients. However, future studies are needed to fully assess the clinical benefit of OAI.
